# Deep Brain Stimulation for Tourette’s Syndrome: The Case for Targeting the Thalamic Centromedian–Parafascicular Complex

**DOI:** 10.3389/fneur.2016.00193

**Published:** 2016-11-10

**Authors:** Paola Testini, Hoon-Ki Min, Asif Bashir, Kendall H. Lee

**Affiliations:** ^1^Department of Neurosurgery, Mayo Clinic, Rochester, MN, USA; ^2^Department of Physiology and Biomedical Engineering, Mayo Clinic, Rochester, MN, USA; ^3^Department of Radiology, Mayo Clinic, Rochester, MN, USA; ^4^Department of Neurosurgery, JFK New Jersey Neuroscience Institute, Edison, NJ, USA

**Keywords:** Tourette, tics, DBS, centromedian–parafascicular, CM-Pf, thalamus

## Abstract

Tourette’s syndrome (TS) is a neurologic condition characterized by both motor and phonic tics and is typically associated with psychiatric comorbidities, including obsessive-compulsive disorder/behavior and attention-deficit hyperactivity disorder, and can be psychologically and socially debilitating. It is considered a disorder of the cortico–striato–thalamo–cortical circuitry, as suggested by pathophysiology studies and therapeutic options. Among these, deep brain stimulation (DBS) of the centromedian–parafascicular nucleus (CM-Pf) of the thalamus is emerging as a valuable treatment modality for patients affected by severe, treatment-resistant TS. Here, we review the most recent experimental evidence for the pivotal role of CM-Pf in the pathophysiology of TS, discuss potential mechanisms of action that may mediate the effects of CM-Pf DBS in TS, and summarize its clinical efficacy.

## Introduction

Tourette’s syndrome (TS) is a neuropsychiatric disorder characterized by motor and phonic tics with onset during childhood; obsessive-compulsive disease and attention-deficit hyperactivity disorder (ADHD) are comorbidities present in a large subset of patients (1). Deep brain stimulation (DBS) of the centromedian–parafascicular nucleus (CM-Pf) is a therapeutic option for severe, medication-refractory TS (2). Although TS is generally considered a disorder of the basal ganglia (BG), with tics taken as evidence of failure to inhibit motor execution, its pathophysiology extends beyond a dysfunction in the BG circuits involving cortical structures in the motor, limbic, and associative networks (3). It is thought that sensorimotor and limbic circuits are affected in TS, prefrontal and limbic circuits in obsessive-compulsive disorder/behavior (OCD/OCB), and sensorimotor, orbitofrontal, and limbic circuits in ADHD (4). The CM-Pf complex, located among the caudal intralaminar nuclei of the thalamus, plays a pivotal role in these networks, providing the principal source of thalamostriatal efferents.

Despite promising preliminary results, CM-Pf DBS for TS is still not approved by the United States Food and Drug Administration and is thus an off-label treatment. A better understanding of the central role of CM-Pf in TS and its treatment can promote further investigation of this therapeutic option and, if efficacy is observed on a larger scale, improve patients’ accessibility to CM-Pf DBS. The rationale for the use of this structure as a target for DBS derives from its importance as the source of thalamostriatal efferents and its major role in functions altered in TS, including attention processing, sensorimotor gating, and motor response (5–11).

Tourette’s syndrome has long been associated with BG dysfunction. The cortico–striato–thalamo–cortical (CSTC) circuits traditionally associated with motor control include two BG pathways, one with facilitatory effects (direct pathway) and one with inhibitory effects (indirect pathway) upon movement. The dopaminergic output from the substantia nigra pars compacta to the striatum upregulates the direct pathway and downregulates the indirect pathway (12). TS has been associated with increased striatal dopaminergic release (13–17), and increased striatal dopamine has been proposed as leading to disinhibition of thalamic output to the cortex, resulting in tics (1). Positron-emission tomography (PET) findings suggest that thalamic DBS modulates dopaminergic circuitries, indicating a likely role for dopamine in therapeutic mechanisms as well (18). Abnormal striatal activity would also lead to the inefficient impulse control associated with OCD/OCB and ADHD, the common comorbidities of TS. In this conception, TS and comorbidities are considered diseases deriving from a failure in the mechanisms responsible for sensory and motor gating (19). In support of the role of BG dysfunction in TS, a recent diffusion-weighted imaging study confirmed that delayed or altered development in the CSTC networks is a possible factor leading to TS manifestations and its normalization is associated with remission of tics (20).

Considering the potential viability of CM-Pf DBS for patients with treatment-resistant TS, a review of the central role of CM-Pf in the complex pathophysiology of TS and its comorbidities is warranted.

## The Role of the CM-Pf in Tourette’s Syndrome

### CM-Pf Connectivity and Function

**Figure 1:**
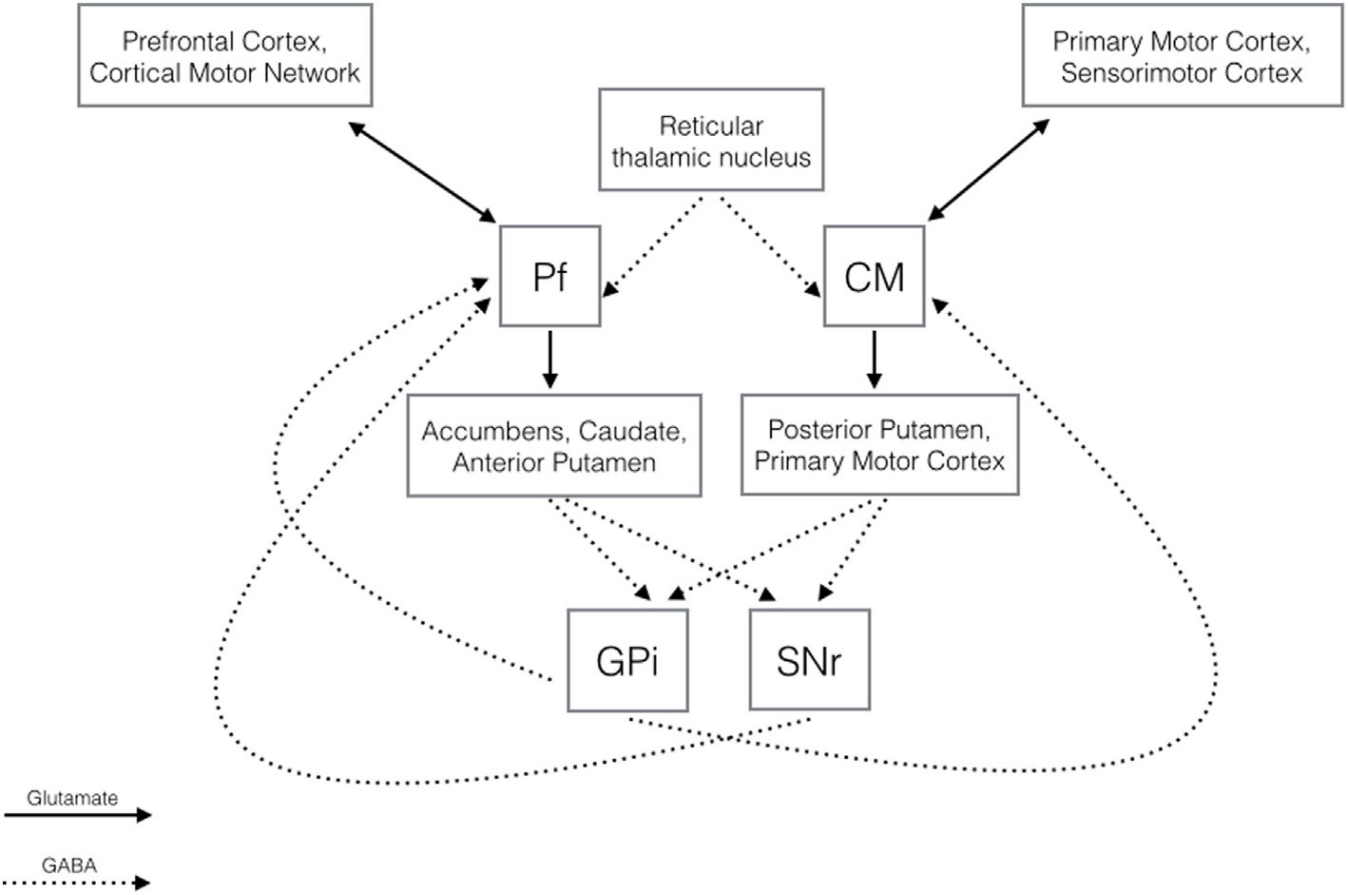
**Diagram illustrating the main GABAergic and glutamatergic connections of the centromedian and parafascicular nuclei (21–25)**.

The hypothesis that the CM-Pf plays a major role in TS is supported by its anatomic and functional connections, which extend beyond motor control to limbic and associative functions. As Figure 1 shows, CM-Pf is densely connected to the BG. The main inputs to the CM-Pf derive from the globus pallidus internus (GPi), and the Pf receives input as well from the substantia nigra pars reticulata. The striatum receives most of the CM-Pf efferents. Although Eckert et al. (10) recently confirmed the extended connectivity between CM-Pf and subcortical structures in a tractography study of normal human subjects, CM-Pf connections have been mainly investigated in studies in rodents and primates, which found that CM (or its rodent equivalent, the lateral Pf nucleus) and Pf have complementary functions.

With its output to the motor cortex and to sensorimotor putamen and afferents from the motor, premotor, and somatosensory cortex, the CM nucleus appears to be involved mainly in motor control. With efferents to the limbic (nucleus accumbens) and associative (caudate, anterior putamen) striatum, anterior cingulate, and premotor and prefrontal cortices, hypothalamus, and amygdala, and input from the prefrontal cortex, supplementary motor area, and frontal eye fields, the Pf plays a role in the limbic and associative network functions. The CM-Pf additionally projects to the subthalamic nucleus, the globus pallidus, the substantia nigra, and the pedunculopontine nucleus and receives afferents from the cerebellum, pedunculopontine nucleus, superior colliculus, brainstem reticular formation, and reticular thalamic nuclei (3, 6, 21–24).

The role of CM-Pf in many functions abnormal in patients with TS, the effects elicited on striatal and cortical activity by manipulations of CM-Pf function, the altered CM-Pf neural activity found in patients with TS, and the intimate connections of the CM-Pf complex with the striatum, a structure pivotal to TS pathophysiology, favor the hypothesis that the CM-Pf plays a central role in TS and its treatment.

The complexity of CM-Pf circuitry reflects the complexity of its functions. As mentioned above, patients with TS often present with a deficiency in sensorimotor gating, as evidenced by the reduction in prepulse inhibition (PPI) of the startle reflex. In rats with decreased PPI, DBS of the medial and lateral Pf normalizes this deficiency (11). Attention deficits are also common in TS (1), and consistent with input from the brainstem reticular formation and reticular thalamic nuclei, the CM-Pf plays a fundamental role in attention arousal (25, 26). The importance of the CM-Pf complex in reward and attention behavior is supported by electrophysiology studies in non-human primates (21, 27, 28), which have confirmed its role in processing sensory stimuli, especially stimuli that are unexpected and unprecedented and thus of high attentional value.

### CM-Pf DBS and Tourette’s

The effects of CM-Pf DBS have been investigated by functional MRI (fMRI) in a large animal model (swine) of CM-Pf DBS (3). The results showed that areas of activation as measured by blood-oxygen-level dependent (BOLD) response varied based on CM versus Pf targeting. BOLD-signal changes in the sensorimotor network (primary motor, premotor, somatosensory cortex) and prefrontal cortex were predominant during CM stimulation, while Pf stimulation induced BOLD-signal changes in limbic regions (including the hippocampus, parahippocampal gyrus, and cingulate cortex). These findings are of relevance given the correlation between tic complexity and altered functional connectivity in the sensorimotor (29) and associative CSTC circuits and between OCD severity and altered functional connectivity in the limbic and associative CSTC circuits (30, 31). Although clinical investigations of the potential differential effects of CM and Pf DBS are needed before conclusions can be drawn, this study suggests that selective modulation of motor, associative, or limbic circuits by DBS of the CM or the Pf may enable more individualized treatment to predominantly target tics versus psychiatric comorbidities in the future. Many of the cortical regions modulated by CM-Pf DBS are structurally and functionally altered in TS (3). The effects of DBS on the activity of supplementary motor, premotor, and prefrontal cortex on the one hand and of the sensory network on the other are of special relevance due to the suggested role of these regions in tic generation and inhibition (32–35), and in PPI and sensorimotor gating, respectively (36).

### Morphologic and Functional Findings in Tourette’s Syndrome

Widespread anatomic and functional neuroimaging abnormalities in the motor, limbic, and associative networks are a feature of TS (37). Sowell and colleagues (38) and Draper and colleagues (39) observed a cortical thinning in the premotor, motor, somatosensory, insular, and anterior cingulate cortices in TS, the severity of which correlated with severity of tics and premonitory urges. This morphological alteration was more prominent in the ventral portions of the sensory and motor homunculi, and the cortical thinning in this area positively correlated with tic severity in the oro-facial district (38). Similarly, cerebellar gray matter volume reductions correlated with tic severity in a recent MRI study performed in patients with TS and healthy controls (40). H_2_O PET found increased brain activity in the prefrontal, primary motor, premotor, supplementary motor, insula, cingulate, cerebellum, thalamus, and striatum correlating with tics (41, 42). Also, fMRI confirmed the increase in activity before tic onset in the limbic (anterior cingulate cortex, insula) and sensorimotor (supplementary motor, premotor, sensory associative cortex, cerebellum) networks, and at tic onset mainly in the sensorimotor network (primary motor, supplementary motor, premotor, somatosensory, sensory association cortex, cerebellum) (43). These findings are in favor of an altered function in the sensorimotor, limbic, and associative networks, with a central role of CM-Pf in modulating the brain areas involved in TS.

Motor network dysfunction has been extensively investigated in TS. PET and transcranial magnetic stimulation studies of patients with TS have found increased resting-state activation, action-related activation, and hyperexcitability in the motor network (44–47). A compromised GABAergic function in the motor system appears to contribute to hyperexcitability in the supplementary motor cortex, as suggested by an altered correlation between GABA levels and beta oscillations in this region (29). The importance of motor network dysfunction in TS is also underscored by the finding that decreased connectivity between the supplementary motor cortex and the striatum correlates with Yale Global Tic Severity Scale (YGTSS) scores (48).

Electrophysiology studies of the CM-Pf target in patients undergoing DBS surgery for TS have found bursting activity in CM-Pf prior to tic production and reduction in the alpha activity and increase in the gamma activity correlated with tic reduction during DBS (49–52). This suggests that CM-Pf DBS may reduce tics by normalizing the local neuronal discharge and affecting striatal output. In fact, *in vivo* stimulation of non-human primate CM nucleus results in GABA-mediated decreased cholinergic activity in the striatum (53), and Pf manipulations (electrical, lesional, or pharmacological) modulate striatal dopaminergic transmission (23).

CM-Pf glutamatergic output exerts a powerful influence over striatal activity, targeting medium-sized spiny neurons and cholinergic interneurons (21). According to one theory of TS pathophysiology, motor pattern generators in the cerebral cortex and brainstem that are associated with a specific movement may be involuntarily activated in TS due to altered regulation of striatal output, leading to tics (54). Given that the striatum is the major target for CM-Pf projections, alterations in this BG structure emphasize the role of CM-Pf in TS.

The major BG morphologic changes associated with TS include reduced volume of both caudate and lenticular nuclei (55–57), and functional imaging of the striatum in TS has shown reduced activity at rest compared to controls (44, 45) and increased activity during tic production (41, 42). Reduced caudate volumes during childhood have been correlated with symptom severity in late adolescence, and larger caudate volumes were found in patients taking neuroleptics for TS compared to the caudate volumes of patients not exposed to these drugs and those of healthy subjects. These findings suggest that a reduced caudate volume may be involved in TS pathophysiology and may be partially due to increased dopaminergic signaling (56, 58).

The reduction in caudate volumes in TS could be mediated by loss, reduced development, or hypofunctioning of GABAergic and tonically active cholinergic interneurons, as suggested by (1) the reduced global striatal expression of genes involved in the steps of interneuron neurotransmission (59); (2) flumazenil-PET findings of reduced GABAergic activity in the striatum (60); and (3) post-mortem findings of reduced GABAergic interneurons in the associative and sensorimotor regions of the striatum (61, 62). In addition, selective inhibition of striatal interneuron activity was associated with dyskinesia and behavioral manifestations of TS in mice (63, 64), indicating that impairment in inhibitory striatal interneuron activity can result in tics.

These observations are at the basis of the hypothesis that the CM-Pf complex is a central node in the modulation of areas and circuitries associated with TS pathophysiology. Despite the elements in support of this theory, DBS remains an invasive treatment and its clinical distribution needs compelling clinical findings before becoming widely accepted and approved as standard of care in selected patients. A summary of available clinical reports of CM-Pf DBS is provided to address the initial translation of the described pathophysiological findings into practice (Table 1).

**Table 1:** **Main clinical outcomes of centromedian–parafascicular deep brain stimulation**.

**Case series/reports**						
**Study**	**Patient number**	**Average (range) percent change Yale Global Tic Severity Scale^a^**	**Average (range) percent change video-based rating**	**Mean follow-up (years)**	**Average (range) percent change Yale-Brown Obsessive Compulsive Scale**	**Adverse events (number of patient affected)**

Vandewalle et al. (65), Visser-Vandewalle et al. (66), Ackermans et al. (67)	3	N/A	−80.9 (−72.2 to −92.6)^c^	5.7	N/A	Reduced energy (3), increased libido (1), reduced erectile and orgasmic function (1), traction pain at the extension cable requiring revision surgeries (2)

Servello et al. (68, 69)	30^e^	−47.0 (range not available)	N/A	2.6	−17.3 (range not available)	Lead repositioning (1), hardware infection requiring system removal (1), surgical wound revision along extension cable due to diasthasis (3), pulse generator pouch infection requiring revision and substitution (2), unilateral extension cable rupture (1), subsequent anterior limb of internal capsule/nucleus accumbens DBS to control OCD (2)

Lee et al. (70)	1	−58.4	−38.5^b^	1.5	N/A	None

Motlagh et al. (71)	5	−50.6 (−7.0 to −85.0) (tic score)	N/A	0.5–8.9	−25.5 (−100 to +35)	Electrode removal because of infection (1) and lack of benefit (1)

Duits et al. (72)	1	−71.4 (stimulation off); −7.1 (stimulation on)	N/A	1.8 (stimulation off); 1.9 (stimulation on)	−60.0 (stimulation off); −65.0 (stimulation on)	Multiple limbs hypertonia, involuntary movements, opisthotonus, impaired consciousness, mutism, impairment of swallowing, nausea, anorexia, death

Idris et al. (73)	1	N/A	N/A	N/A	N/A	Bilateral subcortical hematomas

Savica et al. (74), Testini et al. (75)	10	−53.5 (−12.1 to −100.0); −48.8 (−2.4 to −100.0) (tic score)	N/A	2.2	N/A	Hardware infection requiring surgical wound revision (1)

Bajwa et al. (76)	1	−66.0 (tic score)	N/A	2	−29.0	None

Shields et al. (77)	1	−46.0; −41.0 (tic score)	N/A	0.3	N/A	None

Kaido et al. (78)	3	−34.7 (−29.1 to −43.88); −36.8 (−29.3 to −47.9) (tic score)	N/A	1	+3.7 (+53.8 to −11.5)^d^	None

**Randomized controlled trials^f^**						
**Study**	**Patient number**	**Average (range) percent change Yale Global Tic Severity Scale^a^**	**Average (range) percent change video-based rating**	**Mean follow-up (years)**	**Percent change Yale-Brown Obsessive Compulsive Scale**	**Adverse events (number of patient affected)**

Maciunas et al. (79), Schoenberg et al. (80)	5	−44.0 (range not available); −22.5 (+11.9 to −63.3) (tic score)	−12.5 (+18.8 to −50.0)^b^	0.3	−44.4 (range not available)	Acute psychosis (1), accidental switching off of stimulators with recurrence of tics (2), MVA with recurrence of tics (1)

Okun et al. (81)	5	−18.6 (−5.0 to −30.0); −14.3 (range not available) (tic score)	−25.3 (range not available)^b^	0.5	−5.7	None

Ackermans et al. (82)	6	−49.9 (−26.1 to −94.7) (tic score)	−35.0 (range not available)^b^	1	−33.3 (+100.0 to −100.0)	Parenchymal hemorrhage with vertical gaze palsy (1), lethargy, binge eating, dysarthria, apathy, gait disturbances, falls, cerebral atrophy on CT scan (1), lack of energy (6), subjective visual disturbances (6)

*^a^Total Yale Global Tic Severity Scale (YGTSS) (scale 0–100) if not specified*.

*^b^Modified Rush Video-based Rating Scale*.

*^c^Represent decrease in number of videotaped tics over a 10-min period*.

*^d^One out of three patients experienced an increase in Yale-Brown Obsessive Compulsive Scale (YBOCS) scores; the percent changes for each patient were −11.5, −31.3, and +53.8, respectively*.

*^e^One patient underwent anterior limb of internal capsule/nucleus accumbens lead implantation during the same procedure. The surgical report includes a total of 34 patients of whom 4 were excluded from the analysis [for detail, see Servello et al. (69)]*.

*^f^Score changes reflect the last follow-up reported; one randomized controlled trial (83) was not included in the table because no patient adopted thalamic DBS without pallidal DBS at follow-up*.

*YGTSS, Yale Global Tic Severity Scale; YBOCS, Yale-Brown Obsessive Compulsive Scale; N/A, not available*.

## Clinical Outcomes of CM-Pf DBS for TS

Overall, the outcomes reported for DBS of the CM-Pf for TS have been positive for tic reduction and mixed for psychiatric comorbidities.

### Tic Reduction

The first report of DBS for TS was by Vandewalle et al. (65) who targeted the junction between the CM-Pf complex, the ventrooral internus nucleus (Voi), and the substantia periventricularis (Spv), located 5 mm lateral and 4 mm posterior to the midpoint of the anterior–posterior commissure (AC–PC) line and on the AC–PC plane in a single patient. Long-term results for three patients were later reported by the same group and found that tic reduction, assessed by video recordings, was 92.6% at 10 years (subject 1), 72.2% at 1 year (subject 2), and 78% at 6 years (subject 3) (66, 67). Subsequent series have targeted coordinates at either the anterior border or at the center of the CM-Pf, as discussed below.

The largest series to date (34 patients) of intralaminar thalamus DBS for TS (target located at the CM-Pf/Vo junction, 5 mm lateral and 2 mm posterior to the midpoint of the AC–PC line and on the AC–PC plane) reported statistically significant tic reduction across the group. The average YGTSS scores of the 19 patients who reached the 2-year follow-up significantly decreased from a preoperative average of 76.9 (out of 100) to 36.7, indicating reduction in tics and in disease-related impairment (69). In 17 of these patients, the scores fell from an average of 81.1 preoperatively to 22.5 at the 5- to 6-year follow-up (84). An expansion of this series was recently published, including additional targets (85). As Table 1 shows, results from double-blinded randomized controlled trials and single-case studies or small surgical series tend to support the effectiveness of intralaminar thalamus DBS for TS (70–72, 75, 77–79, 81–83, 86).

### Adverse Events

Adverse events reported in the literature are also summarized in Table 1. Reports from several groups (67, 71, 86–88) have highlighted the potential for increased incidence of complications in DBS for TS, including infections, system breakages, lead traction, and skin dehiscence, possibly associated with self-injurious behaviors, forceful head tics, obsessions, and compulsions, such as scratching associated with the implanted device or the surgical wound and scar.

Of particular note relative to the effects of DBS in patients with severe comorbidities is a patient who participated in the double-blind randomized control study conducted by Ackermans et al. At 23 years of age, she had a history of tics associated with severe self-injurious behavior, pervasive developmental disorder, depression, and compulsions. Following DBS, she developed an array of symptoms and signs (multiple limbs hypertonia, involuntary movements, opisthotonus, impaired consciousness, mutism, impairment of swallowing, nausea, anorexia) suggestive of a psychogenic movement disorder and died in a nursing home (72, 82). Blinded DBS had increased tics and reduced hypertonia. This case highlights the importance of careful evaluation to rule out possible somatoform disorders and treatment of comorbidities prior to DBS surgery. This is of particular relevance given the potential effects of Pf DBS on the limbic and associative networks. The authors did consider alteration of these circuits as a possible cause of postsurgical complications, warning against DBS in case of severe psychiatric comorbidities.

### Psychiatric Comorbidities and Cognition

There is no consensus at this time on whether DBS for TS has a positive, neutral, or detrimental effect on TS-related psychiatric comorbidities and cognition. Results have varied across the literature (67, 68, 70, 71, 76, 78–84, 86, 89, 90).

Considering the complexity of TS and comorbidities, including OCD/OCB, ADHD, depression, and self-injurious behaviors, it is not surprising that in some patients some of these symptoms improve and others deteriorate (67, 82). However, it should be noted that compared to pallidal stimulation, CM-Pf DBS seems to have a more positive impact on depressed mood, emotional hypersensitivity, anxiety, and impulsivity (83, 86). Stimulation of the ventral electrode of CM-Pf leads, which can be considered Pf stimulation, has been associated with feelings of calmness, suggesting a modulation of limbic circuitry (81). This is consistent with the known connectivity of Pf and suggests the need for further investigation of the differential effects of CM and Pf stimulation found in the experimental literature (3).

In the future, large studies may help to elucidate whether the limbic circuitry modulation observed during Pf stimulation exerts positive or detrimental effects on OCD/OCB, ADHD, and depressive symptoms so as to better serve patients whose quality of life is significantly decreased by comorbidities.

### Additional Targets

Other regions have been investigated as possible DBS targets for TS, including the GPi (83, 86, 91–94), the ventroanterior/ventrolateral thalamus (95), the globus pallidus externus (96), the nucleus accumbens and anterior limb of internal capsule (97, 98), and the subthalamic nucleus (99).

The GPi is the most commonly used target after CM-Pf. Results from a recent double-blind randomized crossover trial including 13 patients showed significant tic reduction during GPi stimulation (average 15.3% improvement in YGTSS score during the stimulation-on phase compared to stimulation-off and to baseline phases) (94). Additionally, in a double-blind randomized controlled trial comparing CM-Pf and GPi DBS, stimulation of either target was found to be effective for tic suppression in three patients, with better results obtained with GPi than with CM-Pf DBS (78.3 versus 44.7% reduction in YGTSS scores) (83, 86). Open-label studies including more than ten patients report percent tic score reductions ranging between 44.8 and 52.3, with 59.6% of patients experiencing a YGTSS reduction of 50% or higher (91–93). Further studies comparing CM-Pf and GPi stimulation are warranted to establish a definitive target of choice in the treatment of TS.

### Future Perspectives

As summarized in Table 1, the reports available in the literature display a large range of efficacy outcomes for CM-Pf DBS and of patient population sizes. This is partially related to the small patient group necessitating and being evaluated for DBS treatment and warrants additional investigation to support CM-Pf DBS as a standard therapeutic option in selected patients affected by TS. If multi-center double-blind randomized controlled trials may help achieve larger subject numbers, these are often difficult to implement. Based on current literature characteristics and results, DBS treatment for TS may advance mainly through multiple single-institution, double-blind, randomized controlled studies widely distributed nationally and internationally, which will provide the necessary evidence in support or against the efficacy of this treatment. DBS treatment and patient care will be then performed according to institutional capabilities and therefore not only more feasible but also more closely representative of the following clinical practice outside of randomized controlled studies.

## Conclusion

CM-Pf DBS is a therapeutic option in carefully selected patients affected by severe, treatment refractory TS. CSTC circuitry dysfunction is strongly implicated in the pathophysiology of TS, and the centrality of the CM-Pf to this mechanism makes it a promising target. Larger-scale clinical studies are warranted to confirm the initial promising findings of DBS-related tic suppression. Additionally, the CM appears to play a role in motor functions and the Pf in limbic and associative functions that are associated with TS, and continued investigation of the differential effects of targeting CM for motor symptoms and Pf for psychiatric comorbidities will help determine if more individualized DBS therapy for TS is possible and viable in the future. Large studies investigating the differential long-term outcomes of CM and Pf stimulation for tic control, comorbidities, and cognitive functioning and comparing the available DBS targets for TS, might help DBS for TS transition from an experimental to a more readily available therapeutic option, thus improving the lives of patients with severe, intractable TS and comorbidities.

## Author Contributions

PT: conception and design of the work; interpretation of literature; drafting of the work; final approval of the version to be published; and agreement to be accountable for all aspects of the work. H-KM, AB, and KL: design of the work; critical revision; final approval of the version to be published; and agreement to be accountable for all aspects of the work.

## Conflict of Interest Statement

The authors declare that the research was conducted in the absence of any commercial or financial relationships that could be construed as a potential conflict of interest.
